# *IFITM5*-related (type V) osteogenesis imperfecta with evidence of perinatal involvement: A case report

**DOI:** 10.1016/j.bonr.2024.101766

**Published:** 2024-04-17

**Authors:** Valentina Martínez-Montoya, Miguel Angel Fonseca-Sánchez, Gerardo Fabian-Morales, Ramiro Vega-Gamas, Gloria Eugenia Queipo-García, Luis Felipe León-Madero, Luz María Sánchez-Sánchez

**Affiliations:** a Medical Genetics Service, NanoLab Next Generation Diagnostics, Mexico City, Mexico; b Genetics Service, Instituto Médico de la Visión, Mexico City, Mexico; c Geneticist, Private practice, Mexico; d Genetics Service, Hospital General de México, Mexico City, Mexico; e Kids Doctor, Monterrey, Nuevo León, Mexico

**Keywords:** Osteogenesis imperfecta, *IFITM5*, Prenatal involvement

## Abstract

Osteogenesis imperfecta (OI) is a rare hereditary disorder characterized by bone fragility and frequent fractures. While most cases are attributed to variations in collagen-coding genes *COL1A1* and *COL1A2*, other genes such as *IFITM5* have also been associated with the disease, accounting for up to 5 % of cases. Here, we report a case of a 3-month-old female with a femur fracture and limb deformity. X-rays revealed evidence of osteopenia and previous fractures in the arms, clavicle, ribs, and left limb, alongside prenatal bone deformities detected by ultrasound. Initial clinical evaluation suggested progressively deforming (Sillence's type III) osteogenesis imperfecta (OI). Molecular testing led to the diagnosis of *IFITM5*-related OI, identifying the c.-14C>T (rs587776916) variant. Although this variant has been previously reported in patients with *IFITM5*-related OI, prenatal involvement had not been associated with this variant.

## Highlights

• We observed progressively deforming OI traits in a patient with IFITM5-related OI.
• We identified the c.-14C>T variant, suspecting prenatal manifestations.
• Prenatal manifestations of the c.-14C>T variant have not been previously reported.

## 1 Introduction

Osteogenesis imperfecta (OI) is a genetic disorder of connective tissue characterized by abnormalities in collagen synthesis or processing (Subramanian et al., 2023). Individuals with OI often exhibit a heightened susceptibility to bone fractures due to reduced bone mineral density, earning the colloquial term “brittle bone disease.” (Marini et al., 2007) Core features encompass fractures, bone deformities, and short stature. Nevertheless, extra-skeletal manifestations are also common, including dentinogenesis imperfecta, blue sclera, and hearing disorders (Lindahl et al., 2015). Although variants in *COL1A1* and *COL1A2* genes have traditionally been implicated in up to 90 % of cases of OI, advancements in NGS over the last decade have unveiled a plethora of genes associated with this disease (Lindahl et al., 2015; Unger et al., 2023).

Traditionally, OI has been classified based on clinical and radiological characteristics, with types I - IV associated with the *COL1A1* and *COL1A2* genes, constituting Sillence's classification, which remains widely utilized. These types are autosomal dominant with blue sclerae (type I), perinatal lethal (type II), progressively deforming (type III), and autosomal dominant with normal sclerae (type IV) (Van Dijk et al., 2010). Additionally, type V was later described as a form of OI characterized by calcification in the intraosseous membranes. Recently, with the discovery of new variants associated with OI, more comprehensive classifications and terminologies have been proposed to reflect the diversity of genes associated with this condition (Table S1) (Homan et al., 2011; Van Dijk et al., 2009; Baldridge et al., 2008; van Dijk et al., 2009; Christiansen et al., 2010; Kelley et al., 2011). As various terminologies and classifications are used interchangeably throughout the literature, we will refer to this disorder in a gene-based manner, addressing Sillence's classification as necessary. Readers are encouraged to refer to the supplementary material for a brief review of terminology and nomenclature.

Type V OI, according to Sillence's classification, is caused by a Single Nucleotide Variant (SNV) in the 5′ untranslated region (5’-UTR) of *IFITM5* gene, which anticipates the start codon and elongates the N-terminal region of the bone-restricted IFITM protein (BRIL), also known as IFM5 for the addition of five new amino acids to the wild-type protein (Hanagata, 2016). In addition to variants in *COL1A1* and *COL1A2*, variants in *IFITM5* have been linked to an autosomal dominant inheritance pattern (Forlino and Marini, 2016). Roughly 5 % of all OI cases are attributed to variants in *IFITM5 (*Jovanovic et al., *2022**)*. The phenotype of *IFITM5*-related OI differs from others due to the presence of calcification of the interosseous membrane and the formation of hyperplastic callus. However, this type has a broader phenotypic spectrum than others (Glorieux et al., 2000; Rauch et al., 2013).

In this case report, we present an infant with *IFITM5*-related OI exhibiting a classic severe presentation and no familial history of bone pathology. The patient displayed clinical features consistent with progressively deforming OI (type III according to Sillence's classification) with perinatal manifestations. Sequencing of *IFITM5* uncovered a C>T *de novo* occurring variant in the c.-14 position of the 5’UTR of exon 1, corresponding to rs587776916.

## 2 Subject and methods

### 2.1 Patient report

**Fig. 1:**
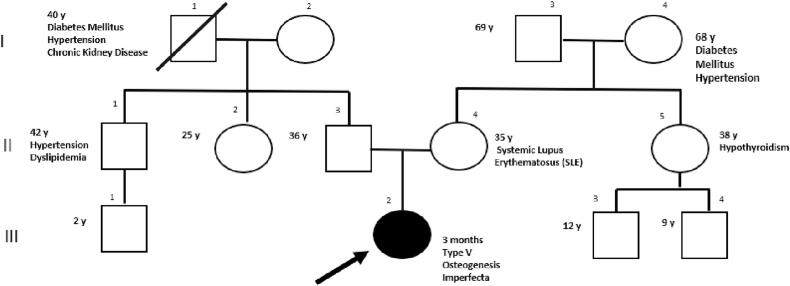
Proband III 2. *IFITM5*-related osteogenesis imperfecta with prenatal involvement. *De novo* gene variant in *IFITM5*. No familial history of bone pathology. Parents are not carriers of the *IFITM5* variant.

The proband, a 3-month-old female patient, was referred to the genetics clinic after experiencing spontaneous fractures. She was the first pregnancy of nonconsanguineous parents of mexican ancestry. Her mother was 34 years old and her father was 37 years old at the patient's birth. Her mother had a clinical history of systemic lupus erythematosus diagnosed at the age of 26, which had been managed with chloroquine, prednisone, and azathioprine. She had been in remission without requiring medical treatment for the past 5 years. Extended familial history included a paternal uncle with hypertension and mixed dyslipidemia, a maternal aunt with hypothyroidism, a paternal grandfather with chronic kidney disease, diabetes, and hypertension (deceased at 40 years of age), a paternal grandmother with hypertension, and a maternal grandmother with hypertension and diabetes. There was no known familial history of bone pathology (Fig. 1).

Her mother received adequate prenatal care, including the intake of multivitamins, iron, and folic acid throughout her pregnancy. However, during week 33, an ultrasound examination indicated moderate oligohydramnios (amniotic fluid index: 8.2 cm). In response, the attending physician advised physical rest and regular fetal monitoring. Subsequent ultrasound scans showed areas of shortening and curvature in the humeri compared to previous images. The patient was delivered *via* cesarean section at 37 weeks of gestation, weighing 2535 g and measuring 45 cm at birth. Extended neonatal screening and auditory testing yielded normal results, and no apparent morphological abnormalities were observed. Further radiological investigations were not deemed necessary.

The patient exhibited irritability and daily crying as reported by the mother. At 3 months of age, an increased volume in the left lower limb above the knee and uncontrollable crying were also observed. The patient was evaluated by a pediatrician, who identified multiple old bone fractures in the upper limbs and left femur. Subsequently, immobilization with a cast was applied to the left leg. The patient was then referred to the genetics clinic, where low weight (5.5 kg, −1.03SD) and height (55 cm, −2.98 SD) were noted. Additionally, the patient exhibited positional plagiocephaly predominantly affecting the right temporoparietal region, blue-gray sclerae, hypertelorism, triangular face, and ulnar deformity of the right forearm. Vital signs were within normal range, and a renal ultrasound revealed no abnormalities. The results of laboratory tests and clinical findings are displayed in Table 1.

**Table 1:** Clinical features and lab test results.

Clinical findings	
• Positional plagiocephaly predominantly at the right temporoparietal region	
• Blue-grayish sclera	
• Anterior auricular pits in the right auricle lobe	
• Hypertelorism, triangular face	
• Ulnar deviation of right forearm	
• Low weight (5.5 kg, −1.03SD) and height (55 cm, −2.98 SD)	

Lab workout (3 months age)	
Value	Normal value or interval
Calcium: 10.3 mg/dL	9–11 mg/dL
Phosphorous: 5.9 mg/dL	3.7–6.5 mg/dL
Magnesium: 2.1 mg/dL	1.5–2.2 mg/dL
Parathormone: 55.48 pg/mL	15–65 pg/mL
25-OH cholecalciferol (vitamin D): 32.14 ng/ mL	30–100 ng/mL
ALP: 935 U/L	< 449 U/L
AST: 57 U/L	< 32 U/L
GGT: 24 U/L	< 33 U/L
Creatinine: 0.21 mg/dL	NA
BUN: 6.5 mg/dL	NA

ALP = alkaline phosphatase; AST = aspartate transaminase; BUN = blood urea nitrogen; GGT = gamma-glutamyl transferase; NA = not available.

**Fig. 2:**
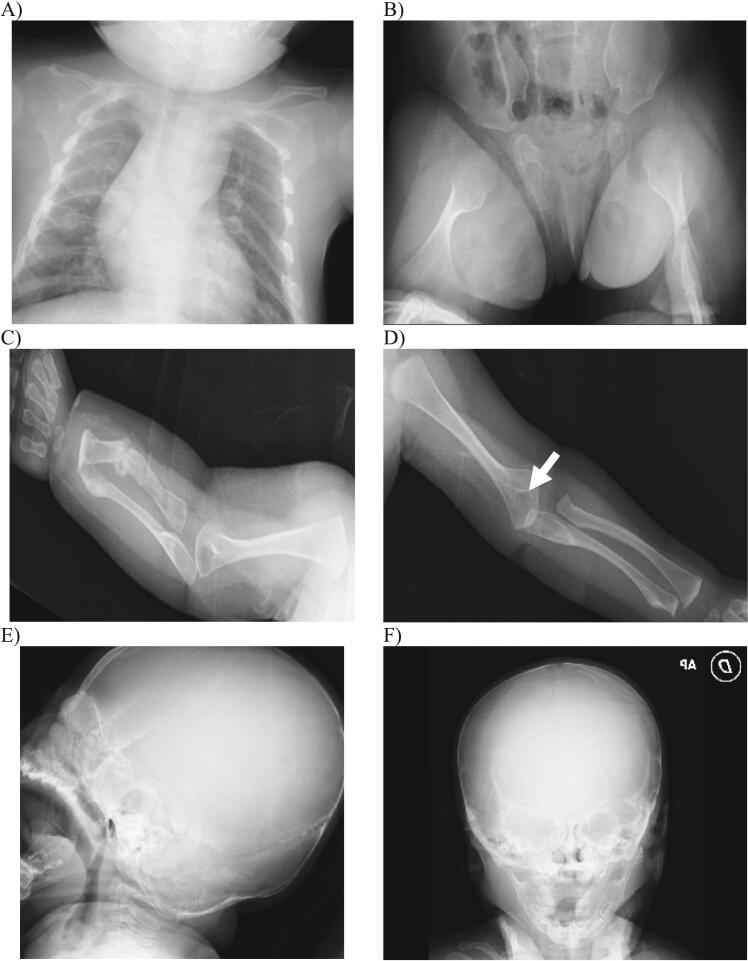
A) Chest X-ray showed callus formation in the 4th, 5th, and 6th left costal arches, as well as the 6th and 7th right costal arches and left clavicle, indicating probable fractures occurring during the prenatal stage. B) Pelvic X-ray revealed a distal metaphyseal fracture of the left femur, angled dorsally at 40°, without involvement of the joint. Pathological widening of the metaphysis was noted, along with overall osteopenia. C) X-ray of the upper right extremity displayed an old diaphyseal fracture of the forearm with bone callus formation. Additionally, a new fracture of the distal radial metaphysis was observed, with the patient wearing a splint at the time of evaluation. D) X-ray of the upper left extremity revealed an old, non-displaced, and non-angulated distal metaphyseal fracture of the humerus. E and F) Cranial X-rays revealed overall demineralization in the diploe, along with Wormian bones in the lambdoid suture.

Radiological tests revealed evidence of overall osteopenia, particularly affecting the long bones, with luminescent bands near the growth cartilages observed in the wrist and left femur. No calcification of the interosseous membranes was identified. Furthermore, an unresolved distal fracture in the left femur and a non-displaced fracture in the left forearm with a bone callus were observed. The presence of bone callus formation observed in X-rays at 3 months old, coupled with ultrasound findings in the third trimester indicating shortened and curved limbs, led to the conclusion that the fractures likely occurred during the pregnancy. Multiple images of old fractures are depicted in Fig. 2A-F.

Based on the phenotype and imaging evidence, progressively deforming (Sillence's type III) OI was suspected. Sequencing of a 24-gene panel associated with OI was requested in order to confirm the diagnosis.

### 2.2 Next-generation sequencing

Parental informed consent was obtained, followed by the extraction and analysis of genomic DNA. Briefly, DNA was extracted with a commercial kit (GeneAll® Biotechnology, Seoul, Korea) and targeted next-generation sequencing (NGS) was performed in an Illumina NextSeq System (Illumina, CA, USA; Agilent Technologies, CA, USA). Variants were evaluated in the exonic and flanking intronic regions (±8 bp) of the probed genes: *ALPL*, *B3GALT6*, *BMP1*, *COL1A1*, *COL1A2*, *CREB3L1*, *CRTAP*, *FKBP10*, *IFITM5*, *LRP5*, *NOTCH2*, *P3H1*, *P4HB*, *PLOD2*, *PLS3*, *PPIB*, *SEC24D*, *SERPINF1*, *SERPINH1*, *SP7*, *SPARC*, *TAPT1*, *TMEM38B*, and *WNT1*. Genome Analysis Toolkit (GATK, Broad Institute, MA, USA) was used for alignment against a reference genome (hg19). 96.4 % of targeted regions were covered at a sequencing depth no <10×, with 94.7 % covered at a sequencing depth no <20×. Results were validated by Sanger sequencing for the proband and parents. Briefly, PCR reactions were conducted using gDNA at a concentration of 1 ng/μL with GoTaq® Flexi DNA polymerase (Promega Corp., WI, USA) following manufacturer's protocol. Primers used for the *IFITM5* gene were: 5′-CAGATGGGATGTCTGTCAGGAG-3′ and 5′-CAGATTCAGGTAGAGGGTGCTG-3. End products were verified by agarose gel electrophoresis.

### 2.3 Patient consent statement

The parents of the patient provided consent for the publication of this clinical case. Following a comprehensive explanation of the publication process, the parents signed an informed consent form specifically for publication purposes.

### 2.4 Statistical analysis

No comparative statistical analysis was conducted as this was a descriptive study.

## 3 Results

**Fig. 3:**
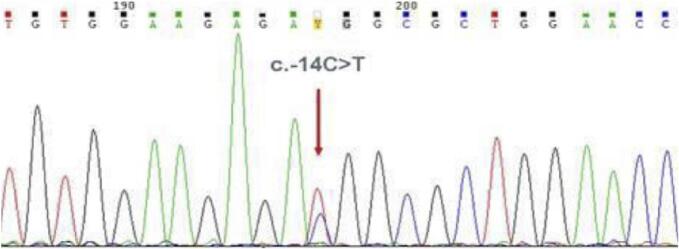
The electropherogram of exon 1 of the *IFITM5* gene demonstrates the substitution of cytosine to thymine (c.-14C>T) in the 5’-UTR region of the gene. This gene variant results in the creation of an alternative upstream start codon within the ORF of *IFITM5*, leading to the production of five new amino acids (Met-Ala-Leu-Glu-Pro) in the N-terminal region of the interferon-induced transmembrane protein 5.

NGS revealed the presence of a heterozygous variant NM_001025295.3: c.-14C>T in the 5' UTR of exon 1 in the *IFITM5* gene corresponding to rs587776916. This variant has been previously associated with *IFITM5*-related OI (corresponding to Sillence's Type V OI, MIM 610967) which exhibits an autosomal dominant inheritance pattern. In accordance with the American College of Medical Genetics and Genomics (ACMG) criteria this variant was classified as probably pathogenic (PM2, PP5). Sanger sequencing of proband confirmed this result (Fig. 3), subsequent analysis of parental DNA revealed a *de novo* occurrence.

## 4 Discussion

*IFITM5* gene belongs to the interferon-induced transmembrane gene family. It is located at the 11p15.5 locus and encodes a protein consisting of 132 amino acids. This protein features two transmembrane domains, N- and C-terminal, along with an intracellular loop. Unlike other genes of the IFITM family, *IFITM5* lacks interferon response elements and is not induced by interferons (Cho et al., 2012). The pathogenic heterozygous variant c.-14C>T (rs587776916) anticipates a start codon which is in-frame with the ORF of the *IFITM5* gene, leading to the addition of 5 new amino acids to the IFITM or BRIL (bone-restricted IFITM-like) proteins. This protein appears to have a crucial role during bone mineralization. The c.-14C>T (rs587776916) variant has been previously reported in the literature as pathogenic associated with type V (according to Sillence's classification) OI (Forlino and Marini, 2016; Rauch et al., 2013; Cho et al., 2012; Grover et al., 2013; Semler et al., 2012).

Murine models of *IFITM5*-related OI have demonstrated a slow rate of mineralization *in utero* and abnormal rib cage formation and long bone deformities and fractures. Notably, gene expression levels in osteoblasts of mutant mice were reduced compared to wild-type mice. Additionally, a reduction in bone mineralization was observed. These results highlight the influence of the c.-14C>T variant on bone formation, contrasting with previous studies that did not observe significant bone involvement in murine models. In an *IFITM5*-related OI mouse model carrying the c.-14C>T variant, perinatal lethality was observed, possibly due to respiratory failure resulting from abnormal rib cage formation. Consequently, it was not possible to determine whether the formation of hyperplastic callus and calcification of the interosseous membrane occurs in the prenatal or postnatal stage (Hanagata, 2016).

Type V (according to Sillence's classification) OI was originally characterized as a distinct OI subtype encompassing hyperplastic callus formation, interosseous membrane calcifications and radioopaque metaphyseal bands in absence of *COL1A1* or *COL1A2* alterations (Glorieux et al., 2000; Rauch et al., 2013). However, these features are not consistent among the patients diagnosed with Type V OI (according to Sillence's classification). For instance, up to 35 % of patients do not exhibit hyperplastic callus. Moreover, phenotype-modifying genes have been proposed as an underlying mechanism of the phenotypic variability observed in these patients (Semler et al., 2012).

This case report portrays a 3-month-old patient with molecular diagnosis of *IFITM5*-related OI caused by a *de novo* c.-14C>T variant (rs587776916). The patient exhibited an intermediate phenotype between those previously described in the literature. Prenatal evidence of upper limb shortening was documented at 35 weeks of gestation. At 3 months of age, a spontaneous fracture in the left femur prompted radiological studies which revealed old fractures and hyperplastic bone calluses, without evidence of calcification of the interosseous membranes of the arms or radial head dislocation. Additionally, the patient exhibited blue-gray discoloration of the sclera. The biochemical bone panel showed an elevation in alkaline phosphatase (ALP), indicating increased bone turnover or growth. Unfortunately, a urinary excretion test for collagen type I N-telopeptide could not be conducted to confirm this suspicion.

*IFITM5*-related OI exhibits a wide phenotypic variability. Patients with clinical features resembling progressively deforming or mild to moderate with normal sclerae OI (type III or type IV according to Sillence's classification) have been reported to be identified as having *IFITM5*-related OI following molecular testing (Grover et al., 2013). This is relevant to our case, as our patient was initially classified as having progressively deforming OI (type III according to Sillence's classification) until molecular testing confirmed the diagnosis of *IFITM5*-related OI.

It is not uncommon to encounter patients who do not exhibit all the radiological findings associated with this subtype, supporting the hypothesis of variable expressivity (Semler et al., 2012). For instance, Grover et al. documented a case of *IFITM5*-related OI where the patient lacked radiological evidence of interosseous membrane ossification in the arms and did not exhibit typical clinical features such as prono-supination alterations, radial head dislocation, and radiodense metaphyseal bands associated with this condition (Grover et al., 2013). In this case, prenatal ultrasound revealing upper limb shortening prompted classification as progressively deforming OI (type III according to Sillence's classification) prior to the availability of molecular diagnosis.

Prenatal involvement of patients with *IFITM5*-related OI has been reported for other variants in this gene. For instance, Gillen Navarro et al. documented a novel variant NM_001025295.3 c.119C>T p.Ser40Leu (rs786201032) in the *IFITM5* gene in a patient exhibiting prenatal limb shortening and the absence of typical clinical features of type V OI. This finding highlighted the lack of allelic heterogeneity in *IFITM5* and raised the suspicion that different variants in *IFITM5* lead to distinct clinical presentations of OI. Additionally, it reported the first case of OI associated with a pathogenic variant in the coding region of exon 1 of *IFITM5* with prenatal involvement (Guillén-Navarro et al., 2014). Hoyer-Kuhn et al. reported a similar case involving the same variant reported by Gillen Navarro in a patient with *IFITM5*-related OI and prenatal involvement (Hoyer-Kuhn et al., 2014). Interestingly, Farber et al. identified the same variant in a 25-year-old woman with severe OI (Farber et al., 2014).

To the best of our knowledge, no cases of *IFITM5*-related OI with prenatal involvement caused by c.-14C>T (rs587776916) variant had been previously reported in the literature. Our patient did not exhibit ultrasonographic evidence of fractures or skeletal dysplasia during the second trimester of gestation, in contrast to the discovery of upper limb shortening during the third-trimester ultrasound, implying that the condition may have developed during this period. Clinical manifestations indicative of OI were evident in our patient within the first three months of life, suggesting that fractures may have occurred prenatally. These observations raise the suspicion of prenatal manifestations of *IFITM5*-related OI associated with the c.-14C>T (rs587776916) variant. However, due to the absence of morphological cues at birth, imaging studies were not initially deemed necessary and were consequently not pursued until three months later when the patient's mother sought medical attention for tenderness in the left lower limb. As such, definitive evidence is currently lacking. During the clinical evaluation, no ossification of the interosseous membrane was documented, suggesting that this phenomenon may occur in later stages of the disease's progression.

This case expands the phenotypic spectrum of *IFITM5*-related OI and strengthens the hypothesis that there may be undiscovered phenotype modifiers linked to pathogenic variants in IFITM5 (Jovanovic et al., 2022; Glorieux et al., 2000; Rauch et al., 2013). Currently, prenatal presentation had been attributed to the c.119C>T p.Ser40Leu variant (rs786201032). However, our case report confirms that c.-14C>T (rs587776916) can also manifest with prenatal presentation, albeit in an advanced stage of fetal development. This observation may be attributed to the dual pathophysiological mechanism observed in several cases of *IFITM5*-related OI, characterized by severe impairment of bone mineralization and exacerbated, defective bone formation. The c.-14C>T variant has previously been associated with defective bone formation, characterized by the presence of bone callus and membrane ossification. This is attributed to a gain-of-function (GOF) mechanism resulting from protein elongation, which contrasts with bone mineralization alterations associated with other variants (Jovanovic et al., 2022). Future studies may elucidate the mechanisms underlying the variability of clinical manifestations of *IFITM5*-related OI.

## 5 Conclusion

*IFITM5*-related OI encompasses a broad spectrum of clinical manifestations. This case further broadens the phenotypic spectrum associated with the c.-14C>T (rs587776916) variant, presenting with spontaneous fractures at 3 months of age and evidence of multiple previous fractures, likely occurring during the prenatal stage. Additionally, the patient exhibited blue-gray discoloration of the sclera and biochemical abnormalities. These findings expand our understanding of the clinical presentations of *IFITM5*-related OI, highlighting the possibility of prenatal manifestations associated with the c.-14C>T (rs587776916) variant.

The following is the supplementary data related to this article.

Table S1 Nosology of osteogenesis imperfecta. Table S1

## CRediT authorship contribution statement

**Valentina Martínez-Montoya:** Writing – review & editing, Writing – original draft, Visualization, Methodology, Investigation, Formal analysis, Conceptualization. **Gerardo Fabian-Morales:** Resources, Methodology, Investigation, Formal analysis. **Miguel Angel Fonseca-Sánchez:** Resources, Methodology, Investigation, Formal analysis. **Ramiro Vega-Gamas:** Resources, Methodology, Investigation, Formal analysis. **Gloria Eugenia Queipo-García:** Resources, Methodology, Investigation, Formal analysis. **Luis Felipe León-Madero:** Validation, Supervision, Investigation, Formal analysis. **Luz María Sánchez-Sánchez:** Writing – review & editing, Validation, Supervision, Project administration, Conceptualization.

## Declaration of competing interest

The authors declare that they have no known competing financial interests or personal relationships that could have appeared to influence the work reported in this paper.

## Data Availability

No data was used for the research described in the article.
