# Supplementary material for: IFITM5-related (type V) osteogenesis imperfecta with evidence of perinatal involvement: A case report
Source: Bone Rep. 2024 Apr 17;21:101766. doi: 10.1016/j.bonr.2024.101766 (PMC11052912; doi:10.1016/j.bonr.2024.101766)
Supplement: Table S1 — Nosology of osteogenesis imperfecta. [file mmc1.docx]

Supplementary Information

| **Table S1.** Nosology of osteogenesis imperfecta | | | | | |
| --- | --- | --- | --- | --- | --- |
| **ID** | **Name of disorder** | **Inheritance** | **Gene or locus** | **MM No.** | **Notes** |
| NOS 26-0010 | Osteogenesis imperfecta, non‐deforming (Sillence type 1), *COL1A1*‐related | AD | *COL1A1* | 166200 | Usually with persistently blue sclerae, can have signs of connective tissue weakness (in MIM as OI type I) |
| NOS 26‐0020 | Osteogenesis imperfecta, non‐deforming (Sillence type 1), *COL1A2*‐related | AD | *COL1A2* | 166200 | Usually with persistently blue sclerae, can have signs of connective tissue weakness (in MIM as OI type I) |
| NOS 26‐0030 | Osteogenesis imperfecta, severe perinatal form (Sillence type 2) *COL1A1*‐related | AD | *COL1A1* | 166210 | Formerly “perinatal lethal”; in OMIM as OI type II |
| NOS 26‐0040 | Osteogenesis imperfecta, severe perinatal form (Sillence type 2), *COL1A2*‐related | AD | *COL1A2* | 166210 | Formerly “perinatal lethal”; in OMIM as OI type II |
| NOS 26‐0050 | Osteogenesis imperfecta, severe perinatal form (Sillence type 2), *CRTAP*‐related | AR | *CRTAP* | 610682 | Formerly “perinatal lethal”; in OMIM as OI type VII |
| NOS 26‐0060 | Osteogenesis imperfecta, severe perinatal form (Sillence type 2), *P3H1*‐related | AR | *P3H1* | 610915 | Formerly “perinatal lethal”; in OMIM as OI type VIII |
| NOS 26‐0070 | Osteogenesis imperfecta, severe perinatal form (Sillence type 2), *PPIB*‐related | AR | *PPIB* | 259440 | Formerly “perinatal lethal”; in OMIM as OI type IX |
| NOS 26‐0080 | Osteogenesis imperfecta, progressively deforming (Sillence type 3), *COL1A1*‐related | AD | *COL1A1* | 259420 | In OMIM as OI type III |
| NOS 26‐0090 | Osteogenesis imperfecta, progressively deforming (Sillence type 3), *COL1A2*‐related | AD | *COL1A2* | 259420 | In OMIM as OI type III |
| NOS 26‐0100 | Osteogenesis imperfecta, progressively deforming (Sillence type 3), *IFITM5*‐related | AD | *IFITM5* | 610967 | In OMIM OI type III; phenotype is distinct but in some  instances can minimize OI type III |
| NOS 26‐0110 | Osteogenesis imperfecta, progressively deforming  (Sillence type 3), *SERPINF1*‐related | AR | *SERPINF1* | 613982 | In OMIM as OI type VI |
| NOS 26‐0120 | Osteogenesis imperfecta, progressively deforming  (Sillence type 3), *CRTAP*‐related | AR | *CRTAP* | 610682 | In OMIM OI type VII |
| NOS 26‐0130 | Osteogenesis imperfecta, progressively deforming  (Sillence type 3), *P3H1*‐related | AR | *P3H1* | 610915 | In OMIM OI type VIII |
| NOS 26‐0140 | Osteogenesis imperfecta, progressively deforming  (Sillence type 3), *PPIB*‐related | AR | *PPIB* | **259440** | In OMIM OI type IX |
| NOS 26‐0150 | Osteogenesis imperfecta, progressively deforming  (Sillence type 3), *SERPINH1*‐related | AR | *SERPINH1* | 613848 | In OMIM OI type X |
| NOS 26‐0160 | Osteogenesis imperfecta, progressively deforming  (Sillence type 3), *FKBP10*‐related | AR | *FKBP10* | 610968 | In OMIM OI type XI |
| NOS 26‐0170 | Osteogenesis imperfecta, progressively deforming  (Sillence type 3), *TMEM38B*‐related | AR | *TMEM38B* | 615066 | In OMIM OI type XIV |
| NOS 26‐0180 | Osteogenesis imperfecta, progressively deforming  (Sillence type 3), *BMP1*‐related | AR | *BMP1* | 614856 | In OMIM OI type XIII |
| NOS 26‐0190 | Osteogenesis imperfecta, progressively deforming  (Sillence type 3), *WNT1*‐related | AR | *WNT1* | 615220 | In OMIM as OI type XV. Biallelic variants; monoallelic variants may result in AD osteoporosis |
| NOS 26‐0200 | Osteogenesis imperfecta, progressively deforming  (Sillence type 3), *CREB3L1*‐related | AR | *CREB3L1* | 616229 | In OMIM as OI type XVI. Has severe joint laxity and scoliosis, Ehlers‐Danlos‐like |
| NOS 26‐0210 | Osteogenesis imperfecta, progressively deforming  (Sillence type 3), *SPARC*‐related | AR | *SPARC* | 616507 | In OMIM as OI type XVII |
| NOS 26‐0220 | Osteogenesis imperfecta, progressively deforming  (Sillence type 3), *TENT5A*‐related | AR | *TENT5A* | 617952 | In OMIM as OI type XVIII |
| NOS 26‐0230 | Osteogenesis imperfecta, progressively deforming  (Sillence type 3), *MBTPS2*‐related | XLR | *MBTPS2* | 301014 | In OMIM as OI type XIX |
| NOS 26‐0240 | Osteogenesis imperfecta, progressively deforming  (Sillence type 3), *MESD*‐related | AR | *MESD* | 618644 | In OMIM as OI type XX |
| NOS 26‐0250 | Osteogenesis imperfecta, progressively deforming  (Sillence type 3) with neurodevelopmental features,  *KDELR2*‐related | AR | *KDELR2* | 619131 | In OMIM as OI type XXI. Frequency of neurodevelopmental delay not clear yet |
| NOS 26‐0260 | Osteogenesis imperfecta, progressively deforming  (Sillence type 3), *CCD134*‐related | AR | *CCD134* | 619795 | In OMIM as OI typeXXII |
| NOS 26‐0270 | Osteogenesis imperfecta, moderate form (Sillence type 4), *COL1A1*‐related | AD | *COL1A1* | 166220 | In OMIM as OI type IV |
| NOS 26‐0280 | Osteogenesis imperfecta, moderate form (Sillence type 4), *COL1A2*‐related | AD | *COL1A2* | 166220 | In OMIM as OI type IV |
| NOS 26‐0290 | Osteogenesis imperfecta, moderate form (Sillence type 4), *WNT1*‐related | AR | *WNT1* | **166220** | In OMIM as OI type XV |
| NOS 26‐0300 | Osteogenesis imperfecta, moderate form (Sillence type 4), *IFITM5*‐related | AD | IFITM5 | 166220 | In OMIM OI type IV |
| NOS 26‐0310 | Osteogenesis imperfecta, moderate form (Sillence type 4), *CRTAP*‐related | AR | CRTAP | **610682** | In OMIM as OI type VII |
| NOS 26‐0320 | Osteogenesis imperfecta, moderate form (Sillence type 4), *PPIB*‐related | AD | PPIB | **259440** | In OMIM as OI type IX |
| NOS 26‐0330 | Osteogenesis imperfecta, moderate form (Sillence type 4), *FKBP10*‐related | AR | FKBP10 | **610968** | In OMIM as OI type XI |
| NOS 26‐0340 | Osteogenesis imperfecta, moderate form (Sillence type 4), *SP7*‐related | AR | SP7 | 613849 | In OMIM as OI type XII |
| NOS 26‐0350 | Osteogenesis imperfecta with calcification of interosseous membranes and/or hypertrophic callus (OI type 5), *IFITM5*‐related | AD | IFITM5 | 610967 | When calcification of intraosseous membranes or hypertrophic callus are not observed, may mimic progressively deforming or moderate OI (Sillence types 3 and 4) |
| NOS 26‐0360 | Osteogenesis imperfecta with craniosynostosis (Cole‐  Carpenter syndrome), *P4HB*‐related | AD | P4HB | 112240 | Craniosynostosis is not well documented in this condition in spite of the name |
| NOS 26‐0370 | Osteogenesis imperfecta with craniosynostosis (Cole‐  Carpenter syndrome), *SEC24D*‐related | AR | SEC24D | 616294 | Was Cole‐Carpenter syndrome 2. Possibly misnomer, as most patients do not have craniosynostosis but rather large fontanels |
| Note: The numbering system (first column) includes “NOS” for “Nosology, skeletal”, followed by the group number and the number of the disorder. The “MIM No.” column shows the MIM number of the disorder; when the number is preceded by “see”, the MIM number is that of the underlying gene. This information was taken from Unger et al 2023 [4]. | | | | | |
